# Synovial sarcoma of the spine in the lumbar vertebral body: A rare case report

**DOI:** 10.1097/MD.0000000000023499

**Published:** 2020-12-11

**Authors:** Qi Feng, Peng Guo, Donglai Wang, Jian Lv, Jiangang Feng

**Affiliations:** a Department of Orthopedics; b Second Department of Surgery, The Fourth Hospital of Hebei Medical University, Shijiazhuang, Hebei, P.R. China.

**Keywords:** lumbar vertebra, soft tissue sarcoma, spine, synovial sarcoma

## Abstract

**Rationale:**

Synovial sarcoma (SS) is a soft tissue neoplasm that rarely occurs in the vertebral body and should be considered in the differential diagnosis in patients with SS and vertebral lesions. SS often presents as a painless mass in the spine, which may undergo slow enlargement, resulting in sustained symptoms of neurologic deficit and pain. Due to the difficulty in differentiating between SS from other soft tissue tumors and metastatic tumors, careful histological confirmation is required for definite diagnosis. Furthermore, due to its malignancy, the appropriate treatment procedure for SS should be carefully considered.

**Patient concerns:**

A 56-year-old female patient had low back pain. Radiological examination revealed bony erosion of the L-2 vertebral body, and no soft tissue mass around the lumbar spine.

**Diagnosis:**

Histopathological and immunohistochemical examination revealed SS.

**Interventions:**

The initial treatment of posterior laminectomy decompression and percutaneous vertebro plasty (PVP) was performed, however, this initial treatment course was inappropriate, but she eventually underwent L-2 complete resection and internal fixation. After the second surgery, she was treated by external beam radiation therapy.

**Outcomes:**

operation radiotherapy was finally performed. No local recurrence in L-2 vertebral body or distant metastasis was found at 1-year follow up postoperation; the neurologic symptom gradually relieved, and no other symptom was noted. And no local recurrence in L-2 vertebral body and distant metastasis was found in 1 year follow up postoperation.

**Lessons:**

Solitary spinal SS is extremely rare. Early surgery for total resection and adjuvant radiotherapy/chemotherapy should be emphasized.

## 1 Introduction

Synovial sarcoma (SS) is a rare malignant tumor. The diagnosis of SS is difficult, and is largely dependent on radiological examination and laboratory examination, including genetic and immunohistochemical examination. Complete surgical resection with negative margins is now regarded as the best treatment. Other treatment options, such as radiation therapy and chemotherapy, are also used However, the prognosis of SS is disappointing.^[1]^ Almost 85% of SSs occur in proximity to the large joints of the extremities and seldom occur elsewhere, especially in the spine.^[2]^ Here, we report a rare case of SS arising in the spine.

## 2 Case representation

**Figure 1:**
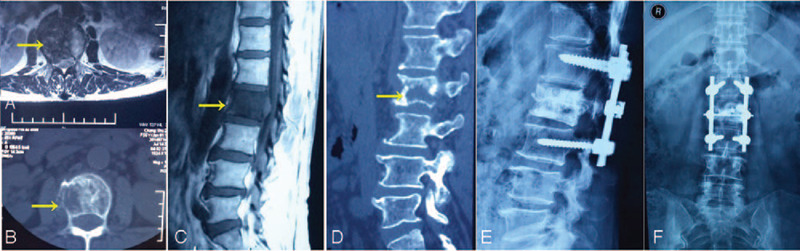
Preoperative magnetic resonance imaging of the lumbar spine before the first surgery (A and C); preoperative computerized tomography imaging before the first surgery (B and D); and postoperative x-ray imaging before the first surgery (E and F).

**Figure 2:**
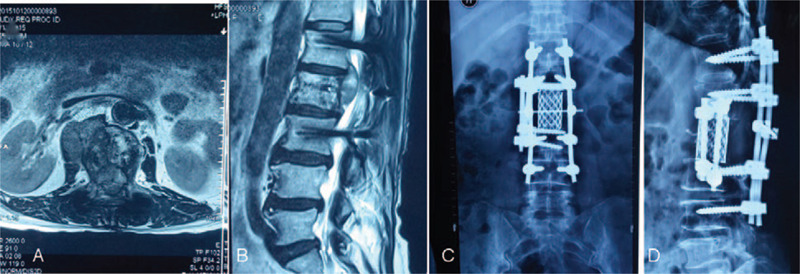
Histopathological and immunohistochemical examination result showing Vim (+), Bcl-2 (+), CD99 (+), CD56 (+), Ki-67 (+), CD117 (–), CK (–), Des (–), EMA (–), S-100 (–), and CD34 (+).

**Figure 3:**
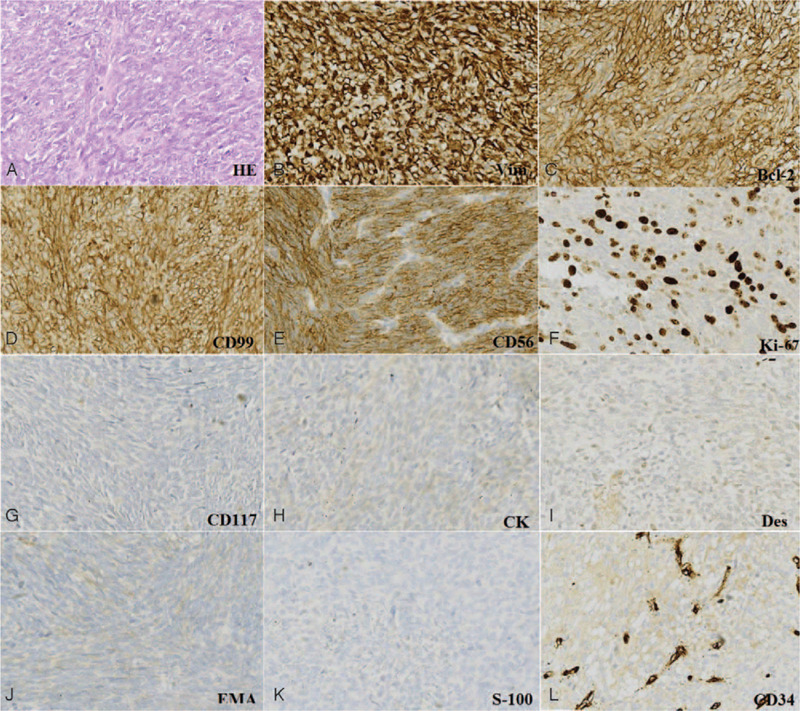
Preoperative magnetic resonance imaging of the lumbar spine before the first surgery (A and B) and postoperative x-ray imaging after the second surgery (C and D).

A 56-year-old woman experienced pain in her waist for >3 months. She occasionally experienced bilateral costal paresthesia and lower extremity or dysfunction and pain in the lower extremities and bladder. The most serious symptom was pain in the lumbar vertebrae, and oftentimes, this was the only symptom. She had no previous trauma or a history of similar symptoms. Radiography revealed hypointense bony erosion of the L-2 vertebral body and no soft tissue mass around the lumbar spine. Computed tomography (CT) revealed multiple circular nodes with homogenous density and clear boundaries in both lungs. Similar to the radiography findings, CT also revealed bony erosion of the L-2 vertebral body, and the density of the adjacent spinal canal was higher than that of the remaining spinal canal (Fig. 1). No evidence of a mass around the spine and no primary or metastatic tumor lesions in other parts of the body were found. No definite evidence of a metastatic lesion was noted on chest radiography and CT. We performed magnetic resonance imaging (MRI) of the thoracic spine (Fig. 1). Core needle biopsy revealed SS of the monophasic type. Histopathological and immunohistochemical examination showed Vim (+), Bcl-2 (+), CD99 (+), CD56 (+), Ki-67 (+), CD117 (–), CK (–), Des (–), EMA (–), S-100 (–), CD34 (+), which was finally diagnosis as SS (Fig. 2), and posterior laminectomy decompression was performed. Because of patients almost suffered the neurological symptom, a midline posterior incision centered on L-2 vertebra was made and the L-2 vertebra and the superior and inferior vertebral articular processes were exposed. Four pedicle screws were inserted, and L-2 vertebra laminectomy and decompression were performed, indirectly reducing operation time. Percutaneous vertebro plasty (PVP) was performed according to the standard PVP procedures (Fig. 1). After operation, the serious symptoms, especially pain, were quickly relieved. She refused to undergo radiotherapy. At the next 1-year follow up, we noted that the neurologic symptom of lower extremity numbness had gradually increased in severity; MRI was performed on day 369 after her first surgery (Fig. 3). The tumor mass was found be enlarged, and spinal cord compression had worsened. After preoperative preparation, the second surgery for lateral total lumbar excision was performed. Titanium mesh combined with an allograft bone was implanted in the space created by L-2 removal, to enable the insertion of a screw for fixation (Fig. 3), after operation radiotherapy was finally performed. No local recurrence in L-2 vertebral body or distant metastasis was found at 1-year follow up postoperation; the neurologic symptom gradually relieved, and no other symptom was noted. And no local recurrence in L-2 vertebral body and distant metastasis was found in 1 year follow up postoperation.

## 3 Discussion

The incidence of SS in soft tissue is relatively low, accounting for nearly 5% to 10% of all soft sarcomas and 1% of all malignancies. Theoretically, SS can occur anywhere in the human body, but the most common primary sites are near the large joints of the upper and lower extremities; >85% of SSs occur at these sites.^[3,4]^ In previous literature, synovial sarcoma of the spine is rare, it occurs in lumbar spine more than other section. Due to the varying appearance of SS, differentiation of SS from other tumors, such as liposarcoma and fibrosarcoma is challenging.^[1]^ Pathologic diagnosis remains the “gold standard.” However, SS found in the spine is simply paraspinal SS, the primary occurrence of SS in the spinal vertebral body is extremely rare. The imaging features of spinal SS also hardly distinguish spinal metastases.^[5]^ Moreover, no special imaging features can be concluded. Due to SS is a malignant tumor, it also presents the behavior of recurrence and metastasis, especially occurs lung metastasis.^[1,6,7]^ In the present case, CT and MRI clearly show that the primary tumor occurred in the L-2 vertebral body.

Consensus on the optimal treatment for SS has not been reached. Complete surgical resection with adjuvant radiotherapy and/or chemotherapy is the currently recommended treatment for SS.^[8,9]^ In the present case, during the first surgery, only bone cement was injected into the vertebral body, and completely excision was not performed. This, in combination with the patient's refusal to undergo radiotherapy/chemotherapy, resulted in poor local tumor control. In contrast to the first surgery, complete tumor excision was achieved during the second surgery and adjuvant radiotherapy was administered, which resulted in a better outcome. This result is similar to that of studies showing that age (≤20 years), tumor size (≤5 cm), a negative surgical margin after complete resection, and the administration of adjuvant radiotherapy are good prognostic factors in patients with SS.^[9]^

In summary, solitary spinal SS is an extremely rare occurrence. Early surgery for total resection and adjuvant radiotherapy should be also emphasized for rare spinal SS to ensure good outcomes.

## Author contributions

**Methodology:** Peng Guo.

**Project administration:** Donglai Wang.

**Writing – original draft:** Qi Feng, Peng Guo, Jian Lv.

**Writing – review & editing:** Jiangang Feng.
